# Anti-oestrogens but not oestrogen deprivation promote cellular invasion in intercellular adhesion-deficient breast cancer cells

**DOI:** 10.1186/bcr2206

**Published:** 2008-12-04

**Authors:** Annabel C Borley, Stephen Hiscox, Julia Gee, Chris Smith, Victoria Shaw, Peter Barrett-Lee, Robert I Nicholson

**Affiliations:** 1 Velindre Cancer Centre, Velindre Road, Cardiff, CF14 2TL, UK; 2 Tenovus Centre for Cancer Research, Welsh School of Pharmacy, Cardiff University, King Edward VII Avenue, Cardiff, CF10 3NB, UK

## Abstract

**Introduction:**

Anti-oestrogens have been the mainstay of therapy in patients with oestrogen-receptor (ER) positive breast cancer and have provided significant improvements in survival. However, their benefits are limited by tumour recurrence in a significant proportion of initially drug-responsive breast cancer patients because of acquired anti-oestrogen resistance. Relapse on such therapies clinically presents as local and/or regional recurrences, frequently with distant metastases, and the prognosis for these patients is poor. The selective ER modulator, tamoxifen, classically exerts gene inhibitory effects during the drug-responsive phase in ER-positive breast cancer cells. Paradoxically, this drug is also able to induce the expression of genes, which in the appropriate cell context may contribute to an adverse cell phenotype. Here we have investigated the effects of tamoxifen and fulvestrant treatment on invasive signalling and compared this with the direct effects of oestrogen withdrawal to mimic the action of aromatase inhibitors.

**Methods:**

The effect of oestrogen and 4-hydroxy-tamoxifen on the invasive capacity of endocrine-sensitive MCF-7 cells, in the presence or absence of functional E-cadherin, was determined by Matrigel invasion assays. Studies also monitored the impact of oestrogen withdrawal or treatment with fulvestrant on cell invasion. Western blotting using phospho-specific antibodies was performed to ascertain changes in invasive signalling in response to the two anti-oestrogens versus both oestradiol treatment and withdrawal.

**Results:**

To the best of our knowledge, we report for the first time that tamoxifen can promote an invasive phenotype in ER-positive breast cancer cells under conditions of poor cell-cell contact and suggest a role for Src kinase and associated pro-invasive genes in this process. Our studies revealed that although this adverse effect is also apparent for further classes of anti-oestrogens, exemplified by the steroidal agent fulvestrant, it is absent during oestrogen withdrawal.

**Conclusions:**

These data highlight a previously unreported effect of tamoxifen (and potentially further anti-oestrogens), that such agents appear able to induce breast cancer cell invasion in a specific context (absence of good cell-cell contacts), where these findings may have major clinical implications for those patients with tumours that have inherently poor intercellular adhesion. In such patients oestrogen deprivation with aromatase inhibitors may be more appropriate.

## Introduction

Despite the undoubted benefits that endocrine therapies have brought for breast cancer patients in terms of increased survival, *de novo *and acquired resistance to such treatments presents a major clinical problem; not all patients with oestrogen-receptor (ER) positive disease benefit and a significant number of initially-responsive patients ultimately relapse on such treatments [1]. The selective ER modulator tamoxifen has been the mainstay of therapy for almost two decades, and much has been learned about acquired resistance to this anti-oestrogen. To date, mechanistic studies have revealed important roles for growth factor signalling pathways such as those regulated by the epidermal growth factor receptor (EGFR) and human epidermal growth factor receptor (HER) 2, as contributors to endocrine resistance [2]. Significantly, in addition to antagonising oestrogen (E2)-regulated gene expression, tamoxifen can promote the re-expression of E2-repressed genes and, importantly, regulate the expression of a unique subset of E2-independent genes [3].

The consequences of such events are only now becoming clear, with recent data suggesting that the ability of selective ER modulators, such as tamoxifen, and the steroidal anti-oestrogen, fulvestrant, to induce expression of signal transduction genes normally repressed by oestrogen/ER signalling may play an important role in the ability of breast cancer cells to evade their growth inhibitory effects [4,5]. Moreover, such treatments may modulate the expression of genes associated with an adverse cell behaviour; for example, in ER-positive breast cancer cells, tamoxifen has been reported to increase expression of 14-3-3, a marker of poor prognosis in breast cancer patients [6].

In addition to their genomic effects, selective ER modulators may also exert non-genomic effects on target cells; for example, tamoxifen has been demonstrated to induce activation of mitogen-activated protein kinase (MAPK) [7], focal adhesion kinase (FAK) [8] and Src [8,9], signalling elements frequently linked to tumour migration and invasion [10,11]. Interestingly, Src kinase is also implicated in limiting the response of tamoxifen, where it stimulates the weak AF-1 function of the tamoxifen-ER complex through its tyrosine kinase activity [12]. Furthermore, in 3Y1 rat fibroblasts, which overexpress Src kinase, tamoxifen cooperates with Src to cause cellular transformation through induction of DNA synthesis and anchorage-independent cell proliferation [13].

E-cadherin is an intercellular adhesion protein important for maintenance of cell-cell adhesion and tissue integrity [14] and much evidence links alterations in its expression with the advent of invasive growth in epithelial tumours [15]. The functional disruption of E-cadherin using monoclonal antibodies can promote Src-dependent cellular invasion [16] and conversely, Src activation has been demonstrated to be essential to the progression of early diffuse gastric tumours, where it is associated with a loss of E-cadherin and the development of local invasion [17]. Significantly, in breast cancer cells, the presence of a functional ER has been shown to be necessary for expression of E-cadherin [18], thus suggesting that its expression and subsequent cell-cell adhesion, may be modulated by anti-oestrogens and may have a bearing on the invasive growth of tumours.

In the current report, we propose a further mechanism that may provide a route for progression of breast cancer in the face of ER inhibition with anti-oestrogens. We have studied the effects of tamoxifen on MCF-7 cells deficient in E-cadherin-mediated homophilic interactions and demonstrate that in a cellular context of reduced intercellular adhesion, tamoxifen promotes an invasive cell phenotype through a process involving the activation of Src kinase. Although also noted with the steroidal anti-oestrogen fulvestrant, this effect of invasion in tamoxifen treated cells was not seen under conditions of oestrogen withdrawal. This evidence suggests that, *in vivo*, a loss of E-cadherin may predict a poor patient outcome on tamoxifen therapy, and may account for some of the additional benefits seen with aromatase inhibitors.

## Materials and methods

### Cell culture

Tamoxifen-responsive, wild-type MCF-7 cells (wtMCF-7) were routinely cultured in RPMI medium (Invitrogen, Paisley, UK) supplemented with 5% foetal calf serum (FCS), antibiotics (10 IU/ml penicillin and 10 μg/ml streptomycin), 2.5 μg/ml fungizone, 200 mM glutamine and incubated at 37°C in a 5% carbon dioxide atmosphere. For experimental analysis, the medium was changed to experimental medium, which is oestrogen depleted, (phenol-red-free RPMI containing 5% charcoal-stripped, steroid-depleted FCS, glutamine and antibiotics as above) for 24 hours before undertaking the relevant assays as described below. These experimental conditions were maintained for oestrogen withdrawal studies, although for anti-oestrogen and hormone treatments the culture medium was supplemented with 10^-7 ^M 4-hydroxy-tamoxifen ('tam'), 10^-9 ^M oestrogen ('E2') or 10^-7 ^M fulvestrant ('Faslodex', AstraZeneca, UK). All tissue culture media and constituents were obtained from Life Technologies Europe Ltd (Paisley, UK) and tissue culture plasticware was obtained from Nunc (Rosklide, Denmark).

### Antibodies and reagents

The antibodies used were as follows: anti-phospho Src kinase (Y418) from Cell Signalling Technologies (New England Biolabs, Herts, UK); pan-Src antibody from Biosource (Invitrogen, Paisley, UK); glyceraldehyde 3-phosphate dehydrogenase (GAPDH) antibody from ABCAM (Cambridge UK); an anti-E-cadherin antibody with neutralising activity (HECD-1) was purchased from R & D Systems Ltd. (Oxford, UK). For immunofluorescence microscopy, the anti-E-cadherin antibody, SHE-78, (Invitrogen, UK) was used.

### siRNA-mediated suppression of E-cadherin in MCF-7 cells

E-cadherin protein expression was suppressed by siRNA transfection of MCF-7 cells as follows: SMARTpool siRNA against human E-cadherin gene (CDH-1) was obtained from Dharmacon Ltd (Perbio Science UK Ltd., Northumberland, UK) and used according to the manufacturer's instructions. Briefly, MCF-7 cells were seeded into six-well plates at 5 × 10^5 ^cells/well in antibiotic-free medium with or without anti-hormone where appropriate. After 24 hours culture, the medium was replaced with fresh, antibiotic-free medium or medium containing transfection lipid, 100 nM non-targeting siRNA control or 100 nM SMARTpool siRNA specific for CDH1 (E-cadherin). Cells were assayed for E-cadherin protein expression after 24, 48 and 72 hours post-transfection by Western blotting to confirm protein knockdown. For invasion assays and Western blotting analysis, cells were treated with CDH1 siRNA for 72 hours before performing the experiments in the presence or absence of the agents as detailed.

### Basement membrane invasion assay

Cell invasion was determined using invasion chambers possessing 8 μm porous membranes (BD Biosciences, Oxford, UK) coated with Matrigel (0.4 μg/ml). Cells (treated as above) were seeded into the chambers (10^5 ^cells/well) with or without anti-hormone and 600 μl of medium was added to the outside of the well. Inserts were cultured at 37°C in a tissue culture incubator for 48 hours, after which the non-invasive cells and Matrigel were removed from the inside of the wells with a cotton swab. After fixing the invaded cells with 3.7% formaldehyde, the porous membranes were removed form the invasion chamber using a scalpel blade and mounted onto a glass microscope slide using Vectashield (Molecular Probes, Eugene, OR, US) containing the nuclear stain 4',6-diamidino-2-phenylindole. Cell invasion was quantified by viewing five separate fields per membrane at a magnification of × 20 and counting the number of cells in each field. Data were then plotted at mean cells per field ± SD for a minimum of three independent experiments, each performed in triplicate.

### Cell lysis and Western blotting

After cell cultures were treated as described above, the cells were washed twice with ice-cold PBS and lysed in lysis buffer (50 mM Tris, pH 7.5, 5 mM ethylene glycol tetraacetic acid, 150 mM sodium chloride and 1% Triton X100) containing protease inhibitors (2 mM sodium orthovanadate, 20 mM sodium fluoride, 1 mM phenyl-methylsulfonyl fluoride, 20 μM phenylarsine, 10 mM sodium molybdate, 10 μg/ml leupeptin and 8 μg/ml aprotinin). The lysates were placed on ice for 20 minutes with intermittent mixing and clarified by centrifugation (10 minutes, 13,000 rpm, 4°C).

The concentration of solubilised proteins was then determined using the DC protein assay kit (BioRad, Hemel Hempstead, UK). From these lysates, 50 μg of total protein was separated by SDS-PAGE using 8% gels and transferred to nitrocellulose membranes by electroblotting. Membranes were subsequently blocked with 5% (w/v) milk protein in Tris-buffered saline containing 0.05% Tween-20. Blots were then incubated with primary antibodies as indicated, washed in Tris-buffered saline containing 0.05% Tween-20 and incubated with horseradish peroxidase-conjugated secondary antibodies. An enhanced chemiluminescence system ('West Dura' reagent, Pierce and Warriner Ltd, Chester, UK) was used for subsequent detection of bound antibodies and the blots exposed to X-ray film (Kodak, UK). Blots shown are representative of a minimum of three separate experiments. Each blot was scanned using a densitometer in order to obtain data for statistical analysis.

### Immunofluorescent staining

Cells were cultured on eight-well chamber slides and allowed to reach log phase growth before being treated with CDH1 siRNA ± tamoxifen as above for a further period of 72 hours. Cells were then fixed with 3.7% formaldehyde, permeablised using 0.05% TritonX-100 and blocked with 10% normal goat serum for 30 minutes. Staining for E-cadherin was carried out using 1 μg/ml SHE78 antibody for 60 minutes followed by anti-mouse immunoglobulin (Ig) G:fluorescein isothiocyanate conjugate (Molecular Probes, Ugene, Oregon, USA) at 1:2000 for 30 minutes. Co-staining for actin was performed by incubating the cells with phalloidin (Invitrogen, Carlsbad, California, USA), in PBS containing 1% BSA. Cells were then washed and mounted onto microscope slides using a hard-set mounting medium (Vectashield, Molecular Probes, Invitrogen, Carlsbad, California, USA). Cells were viewed at a magnification of × 63 with an oil-immersion objective and representative cells photographed.

### Immunocytochemical staining

Log-phase cells, grown on glass cover slips in experimental medium, were left untreated (control) or treated with tamoxifen (10^-7 ^M) or oestrogen (10^-9 ^M) for four days. Cells were fixed in 2% paraformaldehyde/vanadate for 20 minutes before being washed three times for five minutes with PBS. After blocking with PBS-Tween (0.02% v/v), cells were incubated in primary antibody overnight followed by a peroxidise-labelled secondary antibody (DAKO enVision system (DAKO UK Ltd, Ely, Cambridge, UK) for one hour. After washing, bound antibodies were detected using DAB substrate with counterstaining using 0.02% methyl green solution. Stained cells were rinsed, air dried and mounted onto glass slides using a xylene soluble mounting medium.

### Cell growth assay

Cells were seeded into 24-well plates before treating with CDH1 siRNA in the presence or absence of tamoxifen and a range of inhibitors, as described, for 72 hours. After this time, wells were gently washed and cells fixed with formaldehyde (3.7% in PBS). The numbers of cells in each well were then determined using a coulter counter. Data was obtained as cell counts (mean of three separate wells) for each treatment, with experiments performed in duplicate.

### Statistical analysis

Statistical significance was determined with paired student's t-test of the data using MiniTab14. Significance was observed at p ≤ 0.05.

## Results

### Tamoxifen, but not E2-withdrawal, promotes invasion of MCF-7 cells in the absence of intercellular contacts

**Figure 1:**
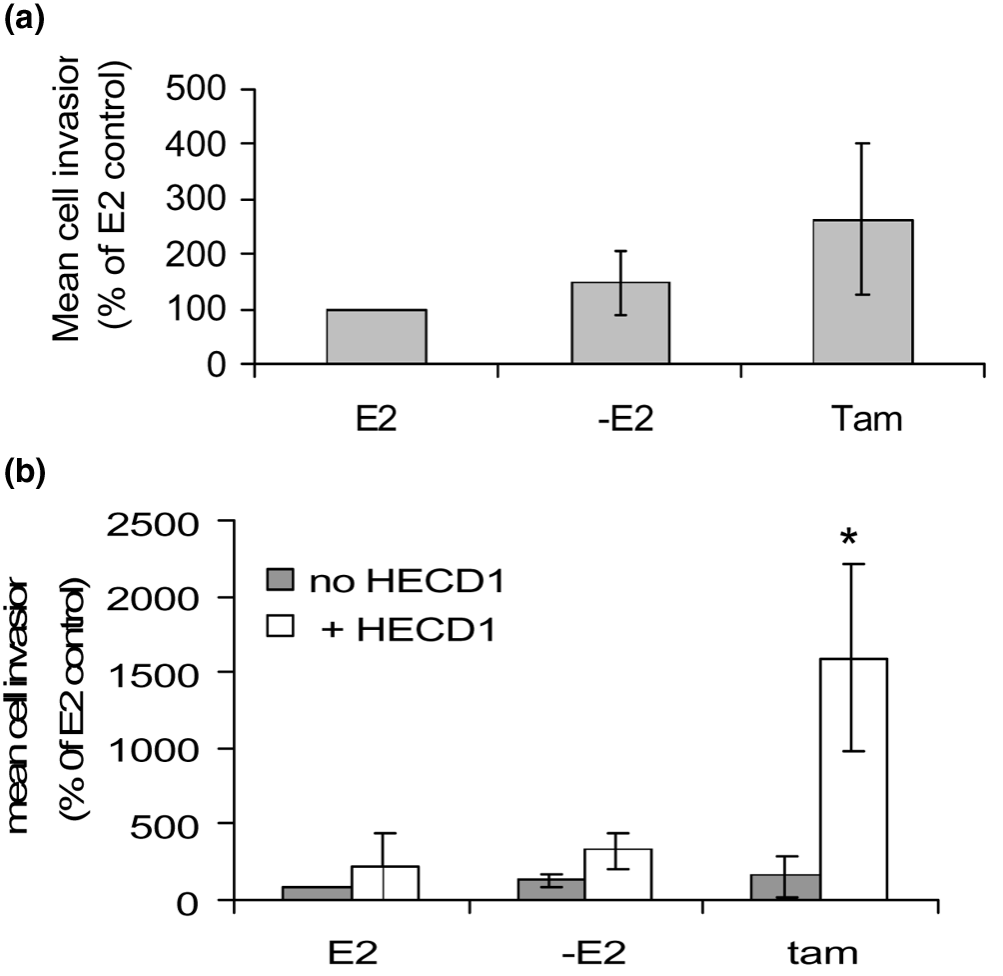
**Tamoxifen induces invasion in HECD-1-treated breast cancer cells**. The invasive capacity of MCF-7 cells having **(a) **functional or **(b) **antibody (HECD-1)-inhibited E-cadherin was determined in the presence of E2 (10^-9 ^M), oestrogen-free (-E2) or tamoxifen-treated conditions (10^-7 ^M). E2 did not significantly alter the invasive ability of MCF-7 cells in either case, whereas **(b) **tamoxifen treatment of HECD-1-treated cells promoted a dramatic increase in invasive capacity. Graphs are representative of three independent experiments with error bars indicating the standard deviation. *p < 0.05 versus treatment with HECD-1 antibody alone.

The capacity of ER-positive, MCF-7 breast cancer cells to invade through Matrigel in response to tamoxifen or oestrogen withdrawal (-E2) was determined and compared with E2 alone. MCF-7 cells are poorly invasive *in vitro *[19] and their invasive capacity was not significantly affected by -E2, while tamoxifen alone had only a minor effect. Interestingly, a non-significant trend emerged, with tamoxifen treatment resulting in increased cell invasion compared with -E2, which in turn caused more invasion than in cells exposed to E2 (Figure 1a).

Whereas neutralising E-cadherin function using the HECD-1 antibody promoted a modest increase in cell invasion, invasion in response to combined treatment (tamoxifen + the antibody) was significantly higher (figure 1b). The use of this antibody in combination with either E2 or under E2-withdrawal conditions ('-E2'), however, did not promote a increase in invasion (Figure 1b). These changes were not due to changes in cell proliferation, because cell proliferation rates were unchanged over the short timescale of the experiment (data not shown).

**Figure 2:**
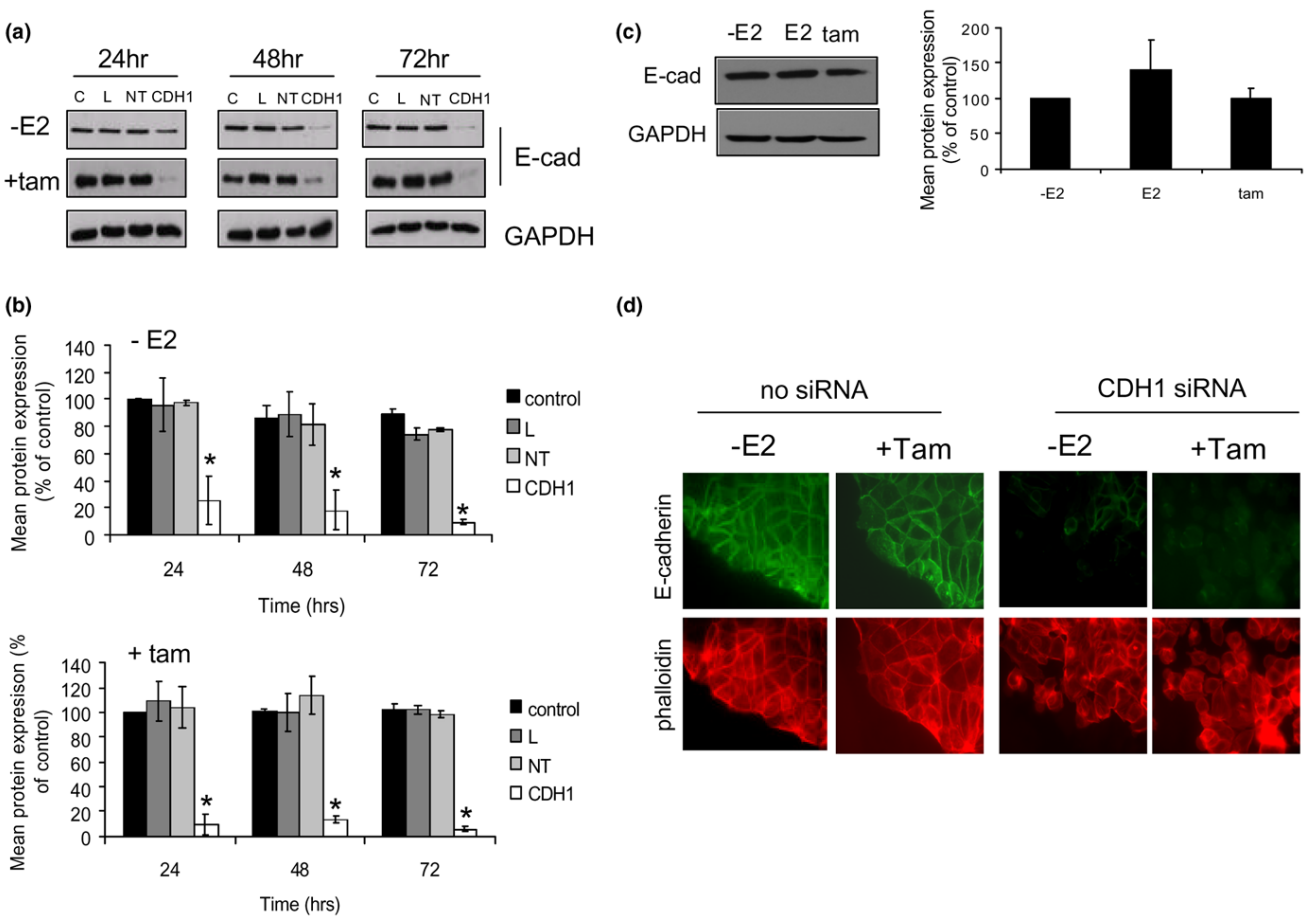
**Modulation of E-cadherin expression in MCF-7 cell using siRNA**. **(a) **MCF-7 cells were left untreated (control, 'C') or treated with transfection lipid ('L'), non-targeting (scrambled) siRNA ('NT') or E-cadherin-specific siRNA ('CDH1') for the times indicated in the presence or absence of tamoxifen. Cells were then lysed and the lysates probed for E-cadherin. **(b) **Densitometry analysis was performed on three separate sample sets and plotted as mean percentage change ± standard deviations *p < 0.05 versus control. siRNA treatment significantly inhibited E-cadherin expression over a period of 72 hours irrespective of the presence of tamoxifen. **(c) **To determine whether E2 or tamoxifen-modulated E-cadherin expression, cells were treated with E2 or tamoxifen, lysed and probed for E-cadherin and β-actin. Neither E2 nor tamoxifen significantly altered E-cadherin expression in these cells. **(d) **MCF-7 cells were treated with plain medium (oestrogen free) or CDH1 siRNA for 72 hours in the presence or absence of tamoxifen before immunofluorescence staining of E-cadherin (green) and actin (red). Cells were then visualised by fluorescence microscopy and representative pictures taken of the same field of view using fluorescein isothiocyanate (FITC) and TRITC (Tetramethyl rhodamine isothiocyanate) filters. Very little or no E-cadherin was detectable after siRNA treatment. Inclusion of tamoxifen alongside the siRNA appeared to increase the numbers of cells having a spherical morphology.

To further confirm that tamoxifen could induce cellular invasion in the absence of intercellular adhesion, we transfected MCF-7 cells with siRNA for the CDH1 (E-cadherin) gene. Cells treated in this way displayed a significant loss of E-cadherin protein, an effect maintained over a period of 72 hours (Figure 2a, b) in contrast to cells treated with either the siRNA delivery lipid or a non-targeting siRNA ('L' and 'NT' respectively). Interestingly, these data appeared to show that tamoxifen increased E-cadherin expression compared with -E2. However, a caveat to this was that these samples were run on separate gels. Thus, to confirm the effects of endocrine manipulation on E-cadherin expression, immunoblots were performed using samples treated with -E2, +E2 or tamoxifen. These data showed that none of the treatments tested had a significant effect on E-cadherin expression (Figure 2c).

siRNA effects were further confirmed with immunofluorescence analysis of E-cadherin expression. Whereas strong staining for E-cadherin could be detected in untreated MCF7 cells (Figure 2d, left two panels), very little or no E-cadherin was observed following 48 hours siRNA treatment (right panels). By co-staining for actin (bottom row), changes in cellular morphology were observed following siRNA-mediated CDH1 suppression. Interestingly, in the samples which were treated with siRNA and tamoxifen combined, the numbers of cells with a more spherical appearance appeared to increase, an event previously linked to enhanced cellular migration [20] (Figure 2d).

**Figure 3:**
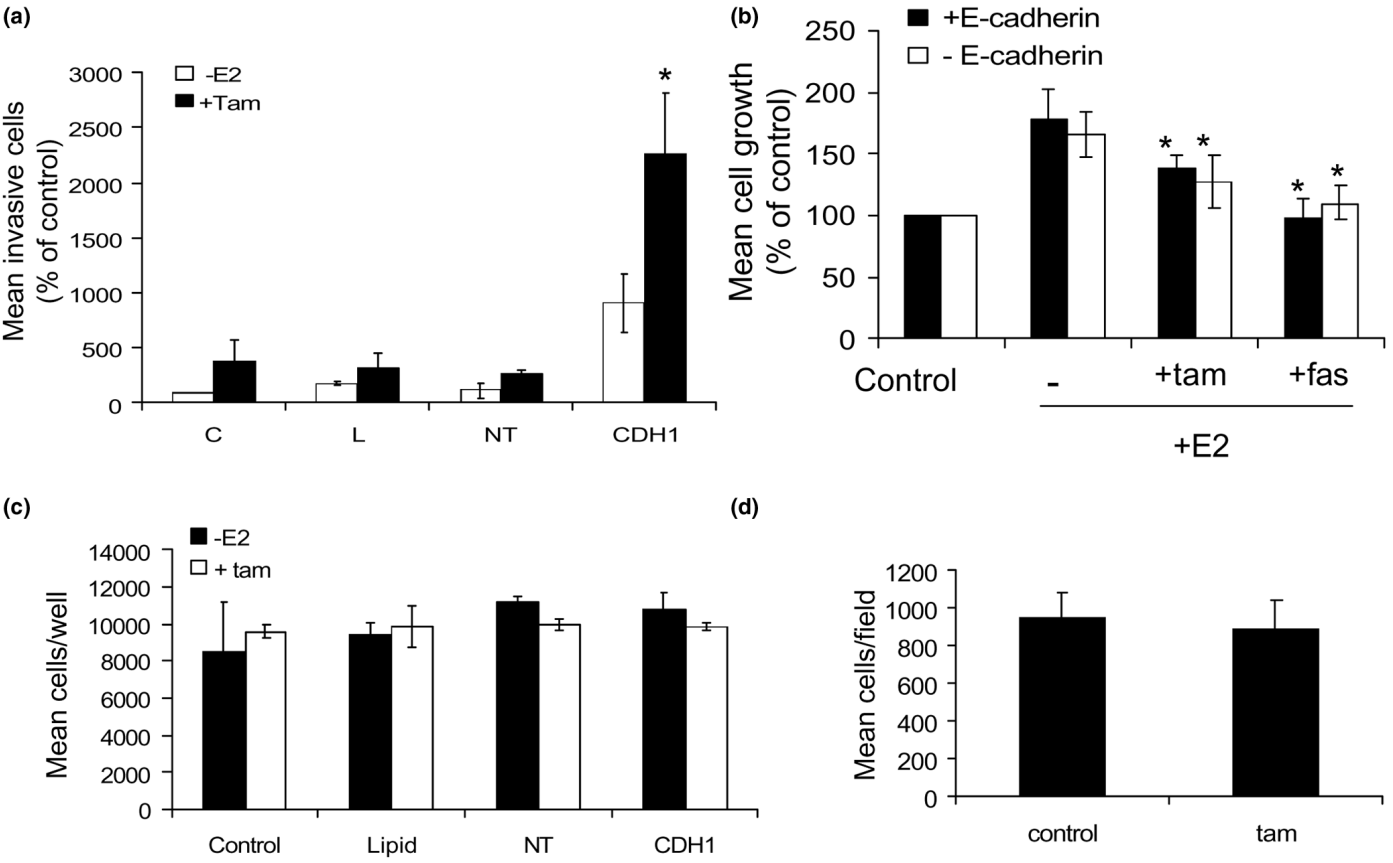
**Tamoxifen promotes invasion of E-cadherin deficient MCF-7 cells**. **(a) **MCF-7 cells were left untreated (control, 'C') or treated with transfection lipid ('L'), non-targeting (scrambled) siRNA ('NT') or E-cadherin-specific siRNA ('CDH1') for 72 hours before assessing their invasive capacity in oestrogen-free and tamoxifen-containing medium. siRNA-mediated suppression of E-cadherin expression alone promoted an increase in cell invasion through Matrigel. Inclusion of tamoxifen in CDH1-treated cells resulted in a further increase in invasive capacity. The graph is the mean of three separate experiments. *p < 0.01 versus CDH siRNA treatment. **(b) **MCF-7 proliferation was determined in response to E2 ± tamoxifen and fulvestrant in the presence and absence of E-cadherin. Data demonstrated that these cells were responsive to both E2 and anti-oestrogens irrespective of E-cadherin presence. *p < 0.05 versus E2 alone. **(c) **The growth of untreated MCF7 cells or cells treated with lipid (L), non-targeting siRNA (NT) or CDH-1 siRNA (CDH1) over five days was determined by coulter counting. No treatment significantly affected cellular growth over this time period. **(d) **MDA-MB-231 cells were treated with siRNA ± tamoxifen and changes in their invasion determined. Tamoxifen did not have any significant effect on MDA-MB-231 cell invasion.

As was the case with the antibody, siRNA-mediated suppression of E-cadherin in the absence of oestrogen resulted in an increase in the numbers of cells invading through the Matrigel basement membrane, although the extent of this was variable (Figure 3a) and overall not significant. However, when tamoxifen was included in this system, the numbers of invading cells was considerably higher (Figure 2a). No effects on invasion were observed following treatment of the cells with either transfection lipid or the non-targeting siRNA, or the addition of E2 (data not shown). Whilst cell growth assays confirmed that these cells were responsive to E2 and antihormones irrespective of E-cadherin expression (Figure 3b), the increases in invasion following antihormone treatment were again not attributable to changes in cellular proliferation as there were no significant changes in cell growth as a consequence of any treatment over the short experimental time period (6 days) (Figure 3c). To determine whether these effects were non-specific and independent of the ER, ER-negative MDA-MB-231 cells were treated similarly. However, no increase in invasion of these cells was seen (Figure 3d).

### Tamoxifen-induced invasion in E-cadherin deficient MCF7 cells involves increases in Src kinase activity

**Figure 4:**
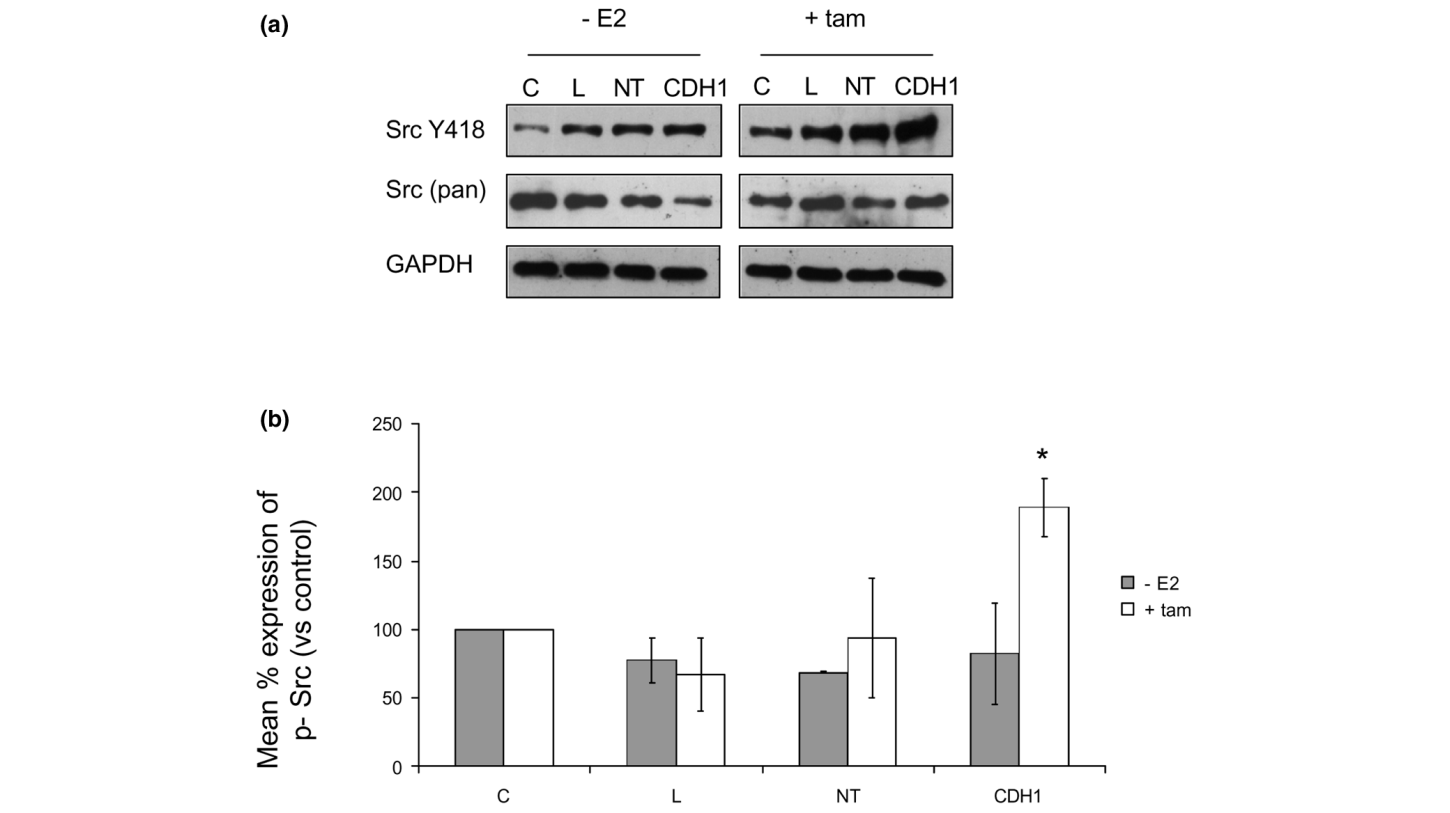
**Tamoxifen induces Src-kinase activity in E-cadherin-deficient MCF-7 cells**. **(a) **MCF-7 cells were treated with plain medium ('C'), transfection lipid ('L'), non-targeting siRNA ('NT') or CDH1 siRNA ('CDH1') for 72 hours before culture in oestrogen-free or tamoxifen-containing medium. Cells were lysed then immunoprobed for activated Src (Src phosphorylated at Y418), total Src (pan Src) and Glyceraldehyde-3-Phosphate Dhydrogenase (GAPDH). **(b) **Replicate immunoprobings were scanned and the data normalised to semi-quantitate the effects of tamoxifen or oestrogen withdrawal on Src activity in the absence of E-cadherin. siRNA-mediated inhibition of E-cadherin combined with tamoxifen caused an increase in the levels of phosphorylated Src detectable in these cells. *p < 0.05 versus untreated cells.

Tamoxifen can activate Src kinase [8] known to promote the invasive phenotype of tamoxifen-resistant MCF-7 cells [21]. Thus, in an attempt to elucidate the mechanism by which tamoxifen may promote invasive behaviour in the absence of intercellular adhesion, we investigated the expression and activity of Src kinase. Src phosphorylation at Y418 was increased in all cells treated with tamoxifen compared with -E2, and was significantly higher in cells treated with the combination of CDH1 siRNA and tamoxifen (Figure 4a, b).

**Figure 5:**
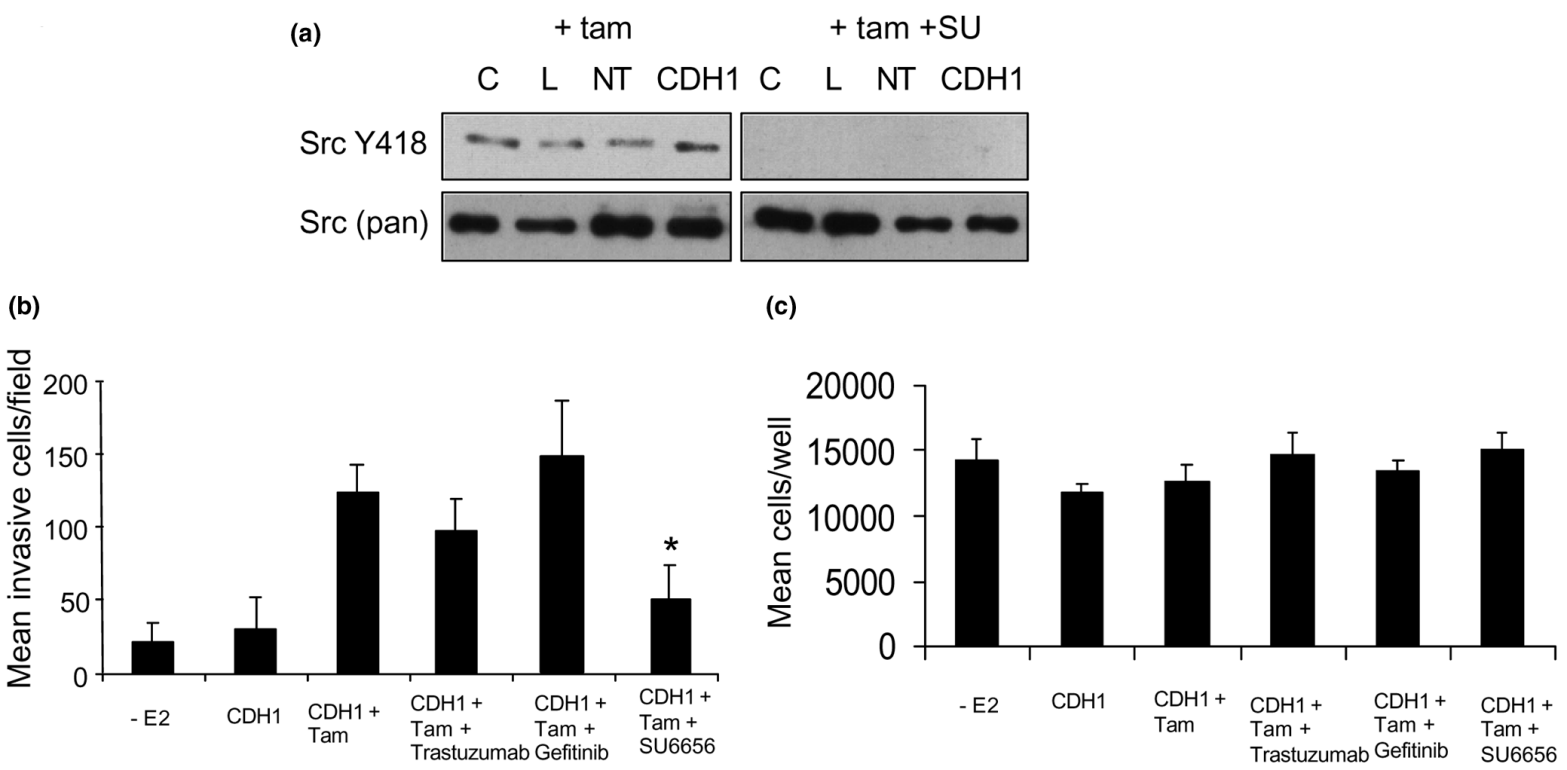
**Inhibition of Src kinase prevents tamoxifen-induced cellular invasion in E-cadherin deficient MCF-7 cells**. **(a) **MCF-7 cells were treated with CDH1 siRNA and tamoxifen in the presence or absence of the Src kinase inhibitor, SU6656 (2.5 μM). SU6656 inhibited Src kinase activity in each of the samples examined. **(b) **MCF-7 cell invasion was determined in E-cadherin deficient MCF-7 cells in the presence of tamoxifen alone, or tamoxifen plus the epidermal growth factor receptor (EGFR) inhibitor, gefitinib ('TKI'), the Src inhibitor, SU6656 ('SU') or the human epidermal growth factor receptor 2 (HER2) inhibitor, herceptin ('Her'). Inhibition of Src activity prevented the tamoxifen-induced invasion in E-cadherin-deficient cells. *p < 0.05 versus cells treated with siRNA and tamoxifen. **(c) **Cell growth in the presence of the same inhibitors was determined by counting the numbers of cells present in the wells of a 24-well plate after 72 hours treatment as shown. No treatment significantly affected cellular growth over this time period.

To determine the functional relevance of elevated Src activity, invasion assays were performed following siRNA and tamoxifen treatment and in the presence of the Src kinase inhibitor, SU6656. Treatment of cells with SU6656 (2.5 μM) inhibited Src kinase activity in all treatment samples while having no effect on total Src levels (Figure 5a). The invasive capacity of these cells was then determined in the presence or absence of SU6656, alongside inhibitors of the EGFR (1 μM gefitinib) and HER2 (100 nM traztuzumab), both previously reported to be upregulated following prolonged tamoxifen treatment in MCF-7 cells [5,22,23]. In these experiments, only inhibition of Src kinase activity was able to significantly suppress the invasion of tamoxifen-treated, E-cadherin-deficient MCF-7 cells (Figure 5b). This was not due to inhibition of cellular growth, as none of the inhibitors tested significantly altered cellular growth over a period of 72 hours, the time period over which the invasion assays were carried out (Figure 5c).

### The steroidal anti-oestrogen fulvestrant also enhances breast cancer cell invasion in the absence of E-cadherin expression

**Figure 6:**
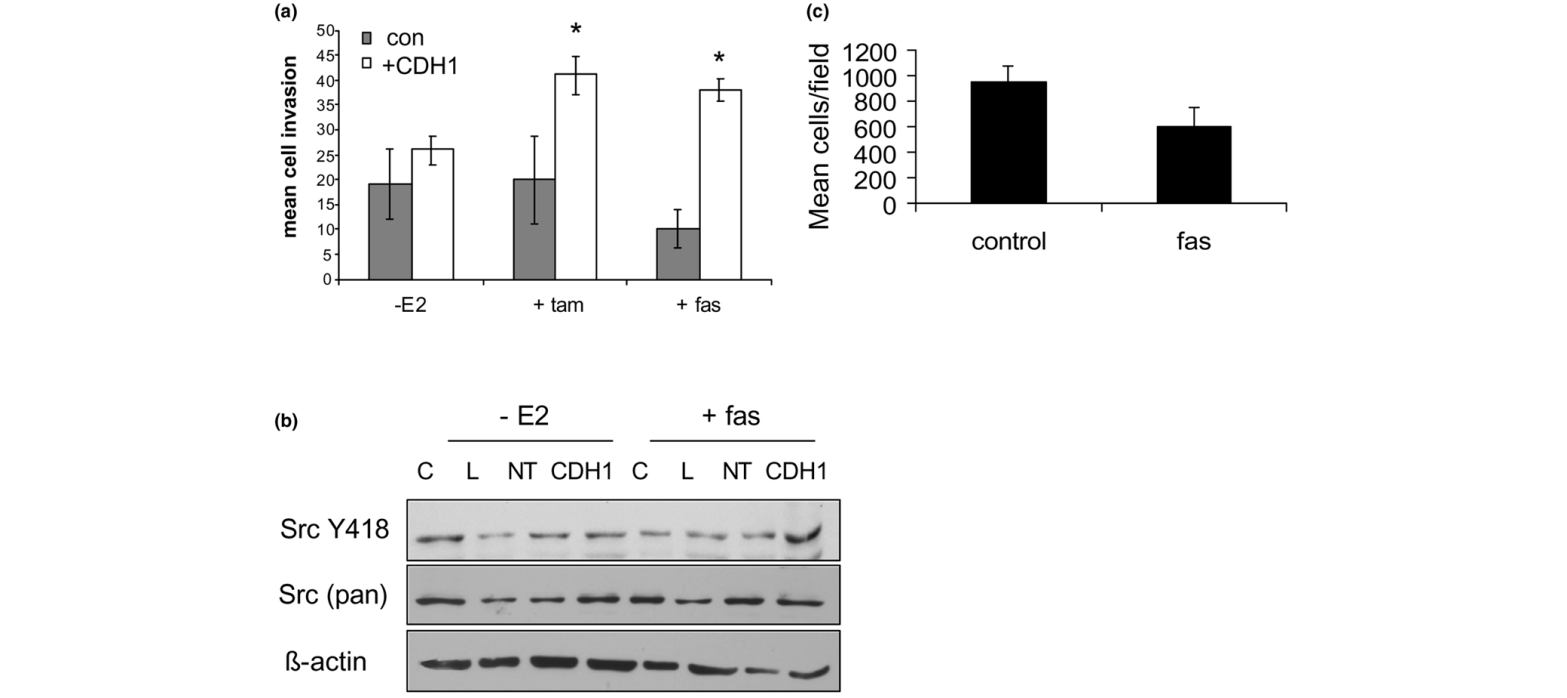
**Fulvestrant enhances breast cancer cell invasion in absence of intercellular adhesion**. **(a) **Fulvestrant also induces invasion in absence of intercellular adhesions, which is accompanied by an **(b) **increase in Src kinase activity. **(c) **No effects are seen in MDA-MB-231 cell invasion following fulvestrant treatment.

To determine whether the invasion-promoting effects of tamoxifen could also be achieved with a different class of anti-oestrogen, experiments were repeated using the steroidal agent fulvestrant instead of tamoxifen. Our data revealed that fulvestrant is also able to induce cellular invasion in the absence of E-cadherin (Figure 6a), whereas oestrogen withdrawal did not. Moreover, in the absence of E-cadherin, only fulvestrant treatment was accompanied by an apparent increase in Src kinase activity (Figure 6b). As was the case for tamoxifen, no gain in invasion was seen after fulvestrant treatment of the ER-negative, E-cadherin-negative MDA-MB-231 cells (Figure 6d).

## Discussion

Adjuvant endocrine therapy has been a major contributor to the decline in breast cancer mortality in the Western world, with the effectiveness of tamoxifen in women with ER-positive cancers clearly demonstrated in many trials [24]. More recently, the dominance of tamoxifen has been challenged by the advent of the aromatase inhibitors, anastrazole, letrozole and exemestane. A number of large randomised trials (e.g. BIG98, MA17 and ATAC [25-27]) have examined the efficacy of aromatase inhibitors in the adjuvant therapy of ER-positive postmenopausal patients, assessing upfront therapy versus tamoxifen, sequential therapy and extended therapy beyond five years. The encouraging results of these studies have led to aromatase inhibitors becoming a standard component of care for these patients. There is also continuing interest in steroidal anti-oestrogens, such as fulvestrant in breast cancer, currently being explored through trials at various stages of the clinical management of the breast cancer patient. However, despite these advances, the optimal endocrine therapy for an individual patient remains uncertain. In addition, a significant number of patients will still experience a recurrence during endocrine therapy and ultimately die of their disease.

It is becoming apparent that administering endocrine agents such as tamoxifen has effects far beyond their initially described mechanism of action and thus attempting to understand the failure of such therapy is a complex problem. Emerging evidence suggests that tamoxifen can modulate cellular processes linked to migratory and invasive responses *in vitro*. For example, tamoxifen can induce FAK-mediated cytoskeletal remodelling [9] and the expression of matrix metalloproteinases [28] by non-genomic mechanisms. Additionally, since the majority of changes in gene expression after oestrogen stimulation are largely thought to be of a repressive nature [3], anti-hormones themselves may promote the re-expression of such genes. Although many of these oestrogen-repressed genes may be growth inhibitory, and thereby contribute to tamoxifen-induced suppression of cell growth, it is increasingly recognised that anti-hormones also induce many genes with an ontology that is not easily reconciled with growth inhibition. Indeed, expression of such genes may contribute towards limiting maximal anti-tumour activity of these agents in ER-positive breast cancer cells [29,30] as is suggested by the ability of tamoxifen and further anti-oestrogens to promote early induction of EGFR and HER2 [5], genes linked to tamoxifen resistance and an adverse cell phenotype.

Furthermore, in the light of reports demonstrating that oestrogens confer a protective effect on invasiveness and motility [31] one might expect anti-hormones to reverse this process. However, despite data from ourselves and others demonstrating a significant, tamoxifen-dependent induction of pro-invasive genes in anti-hormone responsive cells, this does not subsequently translate out into a significant increase in cellular invasive capacity *in vitro*, although a previous study by Mathew and colleagues [32] demonstrated increased healing of MCF-7 cell wounds *in vitro *in response to tamoxifen. Intriguingly, however, our data here indicate the full impact of anti-oestrogen-induced genes may only be manifested under conditions of poor cell-cell contact. Thus, siRNA-induced depletion of E-cadherin-mediated intercellular adhesion, while promoting a modest increase in cellular invasion, greatly enhances the ability of tamoxifen to induce invasive behaviour in MCF-7 cells. Clearly, while this anti-oestrogen confers only small increases in invasiveness under conditions of good cell-cell contact, this may become substantial where cell-cell contact is compromised. These observations are interesting clinically, given that studies have shown that up to 40% of non-lobular breast cancers show reduced or absent E-cadherin expression [33], associated with a poorer prognosis [34,35]. To date, however, it is unknown as to whether E-cadherin status correlates with survival on, or response to, tamoxifen treatment.

Our data suggests that underlying these phenomena is an increase in the activity of Src kinase. Although it has been previously shown that inhibition of E-cadherin function can lead to elevated Src activity [16], our study indicated only a small, non-significant increase in Src phosphorylation following knockdown of the E-cadherin gene. These apparent discrepancies may arise from the fact that in our study, Src activity was monitored after long-term (72 hours) knockdown of E-cadherin expression, compared with short-term (30 minutes), antibody-mediated E-cadherin disruption in the previous case. The effect of tamoxifen on Src activity in this system is interesting in light of the role of Src in cellular invasion. Elevated Src activity is reported in a variety of solid tumours, including breast cancer, and its expression has been shown to increase with disease progression, suggesting an important role in invasion and metastasis [36]. *In vitro *studies have also shown that elevated Src kinase promotes an aggressive and invasive phenotype in tamoxifen-resistant cells [10] and has been linked to the induction of epithelial-to-mesenchymal transition [37,38]. The relevance of Src activity to tamoxifen-induced cellular invasion in our model is further demonstrated by the fact that inhibition of Src phosphorylation significantly reduced tamoxifen-induced invasion in the absence of E-cadherin. The observation that Src inhibition did not completely reverse tamoxifen-induced invasion suggests there are other important mechanisms involved.

Interestingly, the adverse inductive effects do not appear to be specific to the selective ER modulator tamoxifen, because our studies reported here showed that similar invasive responses, and a corresponding increase in Src activity, could also be observed after treatment with fulvestrant (a steroidal anti-oestrogen). Critically, we observed that such events were absent under conditions of oestrogen withdrawal and were not seen in an ER-negative, E-cadherin negative cell line (MDA-MB-231). In total, these data indicate the induction of aggressive cellular behaviour in this model system is a unique consequence of anti-oestrogen occupancy of the oestrogen receptor.

The mechanism by which anti-oestrogens, such as tamoxifen, promote increased Src activity in the absence of E-cadherin is currently unclear. Tamoxifen, itself, can activate signalling pathways via growth factor receptors [39]. Conversely, E-cadherin expression suppresses the activity of receptor tyrosine kinases upstream of Src, such as the EGFR [40]. It is evidenced by the positive regulation of E-cadherin in *in vivo *systems such as the Apc+ mouse facilitating negative regulation of EGFR and associated signalling molecules including Src [41]. Moreover, mice bearing germline mutations in Apc, display impaired E-cadherin-mediated adhesion together with augmented EGFR and Src activity [42]. Thus, the loss of E-cadherin may allow the subsequent activation of growth factor receptors by tamoxifen acting to augment Src kinase activity and leading to an enhanced invasive phenotype. To this end we have investigated the expression and activity of EGFR family members in MCF-7 cells under conditions of E-cadherin deficiency and tamoxifen but have not observed any alteration in expression or activity (A. Borley, unpublished data). However, this does not rule out the role of other growth factor receptors in this process and current studies are underway to address this issue and identify potential candidates.

Our data show that tamoxifen and fulvestrant significantly induce invasion in MCF-7 cells where the E-cadherin intercellular-mediated intercellular adhesion is disrupted, and that activation of Src kinase plays an important role in this process. The clinical implications of these findings are that patients with breast cancers exhibiting aberrant expression in adherens junction components may have a poorer long-term outcome on therapy with a selective ER modulator and that such observations may ultimately extend to steroidal anti-oestrogens. Such changes in expression might be inherent in a subpopulation of breast cancer patients, or induced by long-term endocrine treatment, where prolonged exposure to tamoxifen can promote growth factor pathway activation and the resultant modulation of E-cadherin/catenin function [43]. In such cases, adjuvant tamoxifen therapy may induce expression of pro-invasive genes and promote an adverse cell phenotype given the absence of cell-cell adhesion. We suggest this may lead to an increased risk of aggressive behaviour of recurrences on therapy and hence impact substantially on prognosis. Significantly, our recent studies in an additional ER-positive, E-cadherin-positive breast cancer cell model (T47D cells) also demonstrate that similar pro-invasive effects can be elicited by tamoxifen following suppression of E-cadherin; importantly, this is also accompanied by an increase in Src activity [see Additional data file 1] thus suggesting that these events are not specific to MCF-7 cells, but may represent a generic effect in such breast cancer subtypes.

Importantly, although it might be tempting to speculate that suppression of E-cadherin in MCF-7 cells may represent a model of lobular carcinoma, a subset of breast cancers characterised by low/absent E-cadherin expression [44], this is unlikely because lobular carcinomas are likely to have a multitude of additional changes in gene expression and signalling pathway activity separate from that of a loss in E-cadherin expression. Indeed, a recent report by Rakha and colleagues [45] shows that about 20% of invasive ductal carcinomas have no E-cadherin but are still classed as ductal rather than lobular. Moreover, a number of studies have shown that lobular cancer is a distinct entity of breast cancer that differs from ductal not only in histological and clinical features but also in global transcription programmes [46] and genomic profiles [47].

Lobular carcinomas are generally considered to have more favourable pathological characteristics than ductal carcinomas, including being more likely to express hormone receptors. Although in the early years after diagnosis the prognosis for patients with lobular cancers who receive endocrine therapy appears better than for those with ductal cancers, the overall outcome for lobular cancers may be worse as these show a persistent early relapse rate [45]. Indeed, a recent paper by Pestalozzi and colleagues [48] reports that lobular cancers, despite fairing slightly better than invasive ductal cancers initially, are significantly associated with a reduction in disease-free survival when compared with invasive ductal carcinomas. Such data might suggest that anti-oestrogens may have a beneficial effect on proliferative responses initially but pro-invasive genes expressed over time may promote an invasive phenotype and thus a poor prognosis in cancers lacking E-cadherin. However, no data is currently available concerning the anti-oestrogen-induced expression of pro-invasive genes in lobular cancers. The future availability of *in vitro *cell models of lobular cancer will allow the further examination of anti-oestrogen effects in cells representative of this breast cancer subtype.

Whether tamoxifen-induced cell invasion is a direct result of E-cadherin manipulation or arises from changes within other junction proteins (eg, β-catenin) whose function can be altered after E-cadherin deregulation, or as a result of a loss of cell-cell adhesion *per se*, is unknown at present. We are currently examining a series of clinical breast cancers from the Adjuvant Breast Cancer trial [49] to investigate if E-cadherin (and/or its associated adherens-junction proteins) is a marker of earlier relapse and poorer prognosis on tamoxifen therapy. We suggest further similar studies, correlating E-cadherin expression with outcome, be carried out within the large adjuvant aromatase inhibitor trials.

## Conclusion

Taken together, our pre-clinical data generates the hypothesis that in patients whose primary breast cancers show reduced or aberrant E-cadherin expression, tamoxifen therapy may promote the development of an adverse cell phenotype that may have an impact on disease relapse, its invasive behaviour and hence patient survival. Although our observations may only apply to a relatively small subset of patients, they may account for some of the superiority seen with aromatase inhibitors in the large adjuvant studies. If these findings are borne out, E-cadherin expression could be used a biomarker to help guide the choice of adjuvant endocrine therapy.

## Abbreviations

BSA: bovine serum albumin; ER: oestrogen receptor; EGFR: epidermal growth factor receptor; FAK: focal adhesion kinase; FCS: fetal calf serum; HER2: human epidermal growth factor receptor 2; MAPK: mitogen activate protein kinase; PBS: phosphate buffered saline; siRNA: small interfering RNA.

## Competing interests

The authors declare that they have no competing interests.

## Authors' contributions

SH made the original observations, conceived the study, performed the initial experiments and drafted the manuscript. AB participated in study design, carried out the experimental work, analysed the data and contributed to the manuscript. CS and VS assisted with Western blotting and functional assays. JG, PBL and RIN contributed to study design, interpretation of the results and to the writing of the paper. All authors read and approved the final manuscript.

## Supplementary Material

Additional file 1 Figure that demonstrates the ability of tamoxifen to promote the invasion, and an increase in Src activation, of an additional ER+ cell line, T47D, following E-cadherin knockdown. T47D cells were treated with non-targeting siRNA control (NT control) or siRNA for E-cadherin (CDH1) in the absence of oestrogen (-E2) or presence of tamoxifen (tam) as described for MCF-7 cells. **(a) **Cell invasion was assessed using Matrigel invasion assays while changes in E-cadherin expression and Src activity were determined by **(b) **Western blotting **(c) **with subsequent densitometry. Tamoxifen promoted significant cell invasion in the absence of E-cadherin expression which was accompanied by an increase in Src kinase activity (Src phosphorylated at Y418). Click here for file
