# Supplementary figures and images for: Anti-oestrogens but not oestrogen deprivation promote cellular invasion in intercellular adhesion-deficient breast cancer cells
Source: Breast Cancer Res. 2008 Dec 4;10(6):R103. doi: 10.1186/bcr2206 (PMC2656899; doi:10.1186/bcr2206)

# Supplementary figure 1

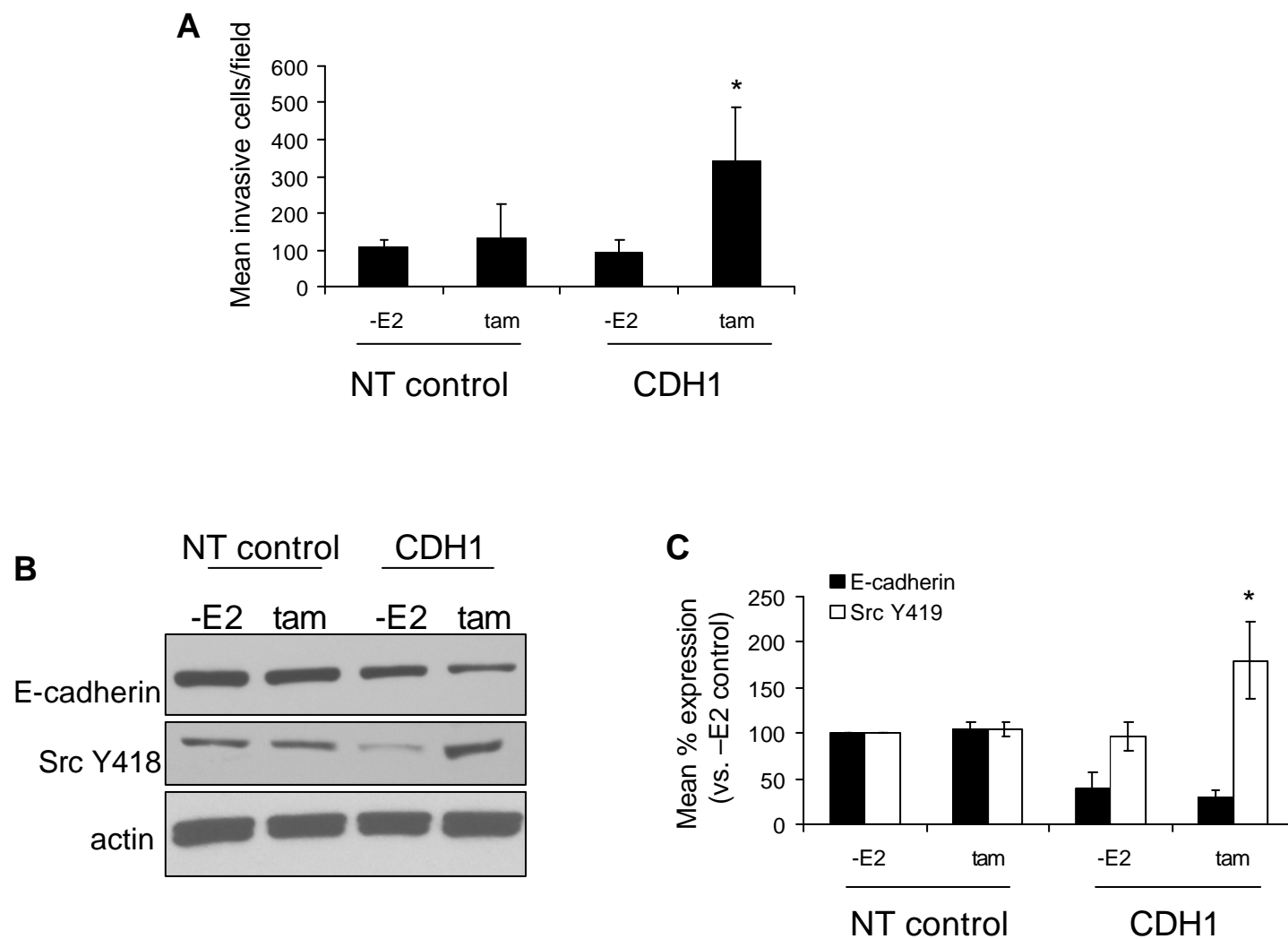

Supplement: Additional file 1 — Figure that demonstrates the ability of tamoxifen to promote the invasion, and an increase in Src activation, of an additional ER+ cell line, T47D, following E-cadherin knockdown. T47D cells were treated with non-targeting siRNA control (NT control) or siRNA for E-cadherin (CDH1) in the absence of oestrogen (-E2) or presence of tamoxifen (tam) as described for MCF-7 cells. (a) Cell invasion was assessed using Matrigel invasion assays while changes in E-cadherin expression and Src activity were determined by (b) Western blotting (c) with subsequent densitometry. Tamoxifen promoted significant cell invasion in the absence of E-cadherin expression which was accompanied by an increase in Src kinase activity (Src phosphorylated at Y418). [file bcr2206-S1.pdf]
